# Eccentric Compression Behavior of Truss-Reinforced Cross-Shaped Concrete-Filled Steel Tubular Columns

**DOI:** 10.3390/ma17153738

**Published:** 2024-07-28

**Authors:** Yu Tao, Sumei Zhang, Gaopeng Xiong, Chao Gong, Zhaoxin Hou, Xiaozhong Li

**Affiliations:** 1 School of Civil and Environmental Engineering, Harbin Institute of Technology, University Town, Shenzhen 518055, China; 19b954014@stu.hit.edu.cn (Y.T.);; 2 Guangdong Provincial Key Laboratory of Intelligent and Resilient Structures for Civil Engineering, Harbin Institute of Technology, Shenzhen 518055, China; 3 Central Research Institute of Building and Construction Co., Ltd., MCC Group, Shenzhen 518055, China

**Keywords:** CCFST, reinforced truss, eccentric compression behavior, ductility, bearing capacity

## Abstract

In the paper, the eccentric compression behavior of the truss-reinforced cross-shaped concrete-filled steel tubular (CCFST) column is investigated. A total of eighteen CCFST columns were tested under eccentric compression, and the key test variables included the reinforced truss node spacing (*s* = 140 mm and 200 mm), slenderness ratio (*λ* = 9.2, 16.6, and 23.1), and eccentricity ratio (*η* = 0, 0.08, and 0.15). The failure mode, deformation characteristic, stress distribution, strain distribution at the mid-span of the steel tube, and the eccentric compression bearing capacity were assessed. The results show that due to the addition of reinforced truss, the steel plates near the mid-span of eccentrically compressed CCFST columns experienced multi-wave buckling rather than single-wave buckling after the peak load was reduced to 85%, and the failure mode of concrete also changed from single-section to multi-section collapse failure. Comparisons were made with the unstiffened specimen. The ductility coefficient of the stiffened specimen with eccentricity ratios of 0.08–0.15 and node spacings of 140 mm~200 mm increased by 70~83%, approaching that of the multi-cell specimens with an increasing steel ratio of 1.8%. In addition, by comparing the test results with the calculation results of four domestic and international design codes, it was found that the Chinese codes CECS159-2018 and GB50936-2014, and the Eurocode 4 (2004) can be better employed to predict the compression bearing capacity of truss-reinforced CCFST columns.

## 1. Introduction

Special-shaped concrete-filled steel tubular (CFST) columns are usually designed in the form of T-shaped, cross-shaped, and L-shaped sections, whose column limbs are aligned with the walls, avoiding the exposure of indoor column corners, facilitating the placement of furniture, improving the efficiency of indoor space utilization, and meeting the functional requirements of residential buildings, which has attracted extensive attention in the engineering field and academia [1,2,3,4,5,6,7]. However, compared with circular or square or rectangular columns [8,9,10,11], traditional special-shaped CFST columns with concave corners tend to cause the separation of the materials, steel tube and concrete, under load. The separation prevents the steel tube from providing effective horizontal confinement to the concrete, thereby undermining the optimal performance of the steel tube and concrete.

To enhance the combined effect of steel tube and core concrete, scholars have proposed a series of methods to stiffen special-shaped CFST columns with the aim of increasing bearing capacity or ductility. One effective approach involves configuring multi-cell sections [2,3,7,12,13,14,15,16]. For instance, Du Guofeng et al. [17] conducted tests on the T-shaped multi-cell CFST members under eccentric compression. The results revealed that the multi-cell arrangement of the T-shaped CFST column could enhance synergistic working, leading to improved mechanical performance. Tu Yongqing et al. [14,15,18] conducted compression tests on T-shaped multi-cell CFST columns. Through the analysis of test data and finite element simulation results, the influence of various parameters on the column’s bearing capacity was examined. Li Quan et al.’s [19] research on eccentric compression tests of T-shaped CFST columns with different slenderness ratios demonstrated that the impact of eccentricity directions (0° and 90°) was relatively minor. Some scholars have also improved the compression behavior of special-shaped CFST columns by adding longitudinal stiffening ribs [3,20,21,22,23], binding bars [24,25,26,27,28,29,30], or tensile bars [31]. For instance, scholars like Zuo Zhiliang, Cai Jian, and Long Yueling [24,25,26,27,28] conducted axial and eccentric compression tests on L-shaped and T-shaped CFST columns with binding bars. They analyzed the impact of horizontal spacings, eccentricity, and load angle on axial and eccentric compression behavior. Yang Yuanlong et al. [23,31] investigated T-shaped CFST columns with longitudinal stiffening ribs and tensile bars stiffener. They conducted axial and eccentric compression tests and conducted finite element analysis to study their bearing capacity and stability. The measures can enhance the synergistic working between the two materials, steel tube and concrete; however, several issues remain, such as more complex processing technology, steel plate sidewall unevenness, an insufficient improvement of component ductility, and a high steel ratio.

To address the above issues, a truss-reinforced CCFST column is proposed [32]. The design ingeniously welds bent bars to the edges of the grooved steel plate, forming reinforced truss within the specimen. The configuration better improves the restraining effect on the sidewalls of the steel plate. The reinforced truss form is characterized by high machining accuracy, high quality welding, and modular production after adopting automated equipment. Meanwhile, the tensile force on the diagonal steel bars is direct, and the effect on the steel plate at the concave corner is obvious. Compared with the multi-cell form, the steel consumption inside the member with reinforced truss is greatly reduced; compared with the binding bar form, the bent steel bars are only welded on the short steel plate extending inside the steel tube, which avoids the problem of opening holes on the plate and unevenness on the outer surface of the steel plate caused by bolts, and at the same time, it avoids the residual stresses on the steel tube generated by multiple welding. Another study [33] investigated the axial compressive mechanical properties of the truss-reinforced CCFST short column, and the results showed that the setting of reinforced trusses can effectively enhance the confinement effect of steel tube on concrete and the ductility of the member was significantly improved. However, the type of member has not been studied under eccentric compression. Therefore, this paper takes the truss-reinforced CCFST column as the research object, and a total of eighteen CCFST columns are tested under eccentric compression. The influence of parameters such as the reinforced truss node spacing, reinforced truss node spacing (*s* = 140 mm and 200 mm), slenderness ratio (λ = 9.2, 16.6, and 23.1), and eccentricity ratio (*η* = 0, 0.08, and 0.15) on the member’s eccentric compression behavior is investigated. The effect on the amount of eccentric compression behavior is also elaborated on the member’s working mechanism under eccentric compression, and relevant suggestions on the calculation of the eccentric compression bearing capacity applicable to the truss-reinforced members are given, aiming at providing references for theoretical analyses and practical engineering applications.

## 2. Test Program

### 2.1. Details of Specimens

**Figure 1:**
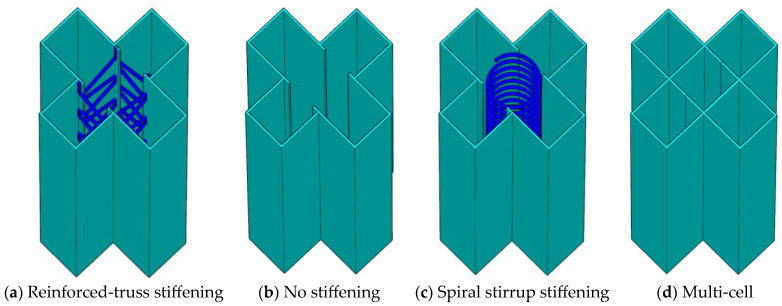
Schematic diagram of specimen structure.

**Figure 2:**
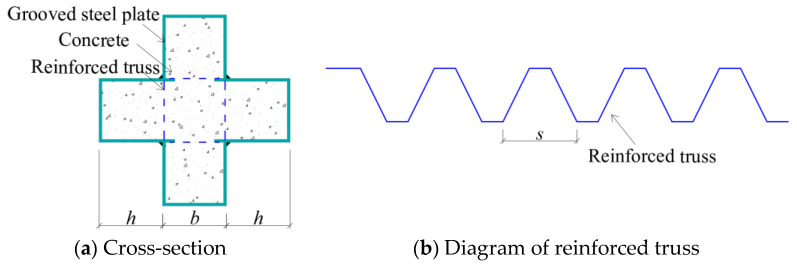
The truss-reinforced CCFST column.

In order to study the eccentric compression behavior of truss-reinforced CCFST columns, 12 truss-reinforced CCFST specimens, 2 unstiffened comparative specimens, 2 spiral stirrup-stiffened comparative specimens, and 2 multi-cell comparative specimens were designed and fabricated, and the configuration forms are displayed in Figure 1. The limb height and limb thickness of all specimens were 120 mm. Concrete with a strength grade of C50 was used, the steel tube was made of steel plate with a thickness of 4 mm and a grade of Q235, and the truss was made of HPB300 steel bar with a diameter of 8 mm (Figure 2). Table 1 lists the parameters of specimens in detail; *α* is the steel ratio; CR and C in the specimen label represent the truss-reinforced specimen and the unstiffened specimen, respectively; and CL and CD represent the truss-reinforced cross-shaped specimen and the multi-cell specimen, respectively. The letters a, b, and c correspond to different slenderness ratios *λ*. The last group of numbers in all specimen labels represents the loading eccentricity *e*, and the reciprocal second group of numbers denotes the reinforced truss node spacing *s* or the spiral stirrup pitch *d*. Taking “CRa-140-27” as an example, “CR” means that the specimen is a truss-reinforced specimen, “a” means that the slenderness ratio *λ* is 9.2, “140” means that the reinforced truss node spacing *s* is 140 mm, and “27” means that the load eccentricity *e* is 27 mm, i.e., the eccentricity ratio is 0.08.

**Figure 3:**
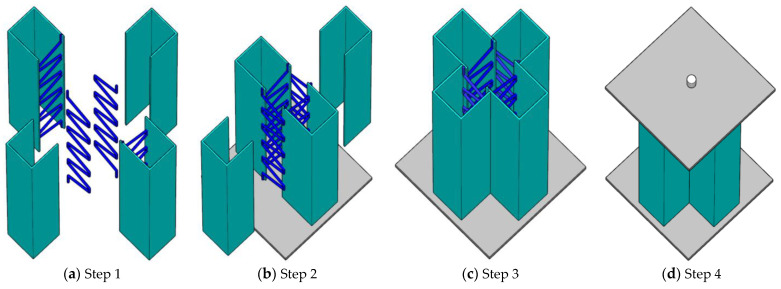
Processing schematic of truss-reinforced CCFST columns.

The fabrication of the specimen was carried out according to the configuration form of the designed truss-reinforced cross-shaped specimens. Firstly, the steel plates were cold-bent into groove shape, the steel bars were bent, and the bent bars were welded to the edges of the grooved steel plate (Figure 3a). The welding of steel plate and steel bar was carried out by CO_2_ gas arc welding with a welding foot height of 5 mm, and continuous welding was carried out within the length (40 mm) of the short steel bar in contact with the steel plate. Secondly, the grooved steel plate was welded to the lower cover plate (Figure 3b), and then two additional grooved plates were welded to the previous plate to form a cross-shaped closed cavity (Figure 3c), and concrete was poured inside the cavity of the cross-shaped steel member. Finally, after 28 days of curing, the concrete on the upper surface of the specimen was polished and smoothed, and the upper cover plate and the hinge were welded to complete the production of the specimen (Figure 3d).

### 2.2. Material Properties

The main mechanical property indexes of core concrete were determined in accordance with the Chinese standard GB/T 50081-2019 “Ministry of Housing and Urban-Rural Development of the People’s Republic of China, Standard for Test Method of Concrete Physical and Mechanical Properties” [36]. The compressive strength *f*_cu_ of the 150 mm × 150 mm × 150 mm cube test block was 44.1 MPa, the elastic modulus *E*_c_ was 32.3 Gpa, and Poisson’s ratio *μ*_c_ was 0.221. The tensile tests for the steel tube and steel bars were determined in accordance with the Chinese standard GB/T 228-2010 “Metallic Materials-Tensile Testing-Part 1: Method of Test at Room Temperature” [37]. Steel bars do not have an obvious yielding platform. Thus, the yield strength is determined by the 0.2% proof stress. The measured mechanical properties of the steel are shown in Table 2, where *f*_y_ and *f*_u_ are the yield strength and tensile strength of the steel tube, respectively, and *E*_s_ and *μ*_s_ are the elastic modulus and Poisson’s ratio of the steel plates, respectively.

### 2.3. Test Setup and Measurement

**Figure 4:**
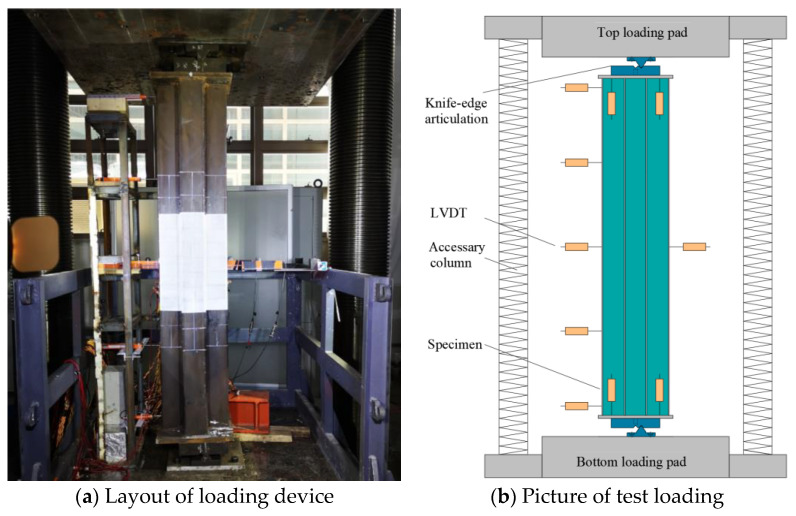
Test setup.

Eccentric compression tests were carried out using a 30,000 kN universal testing machine (Figure 4). The machine came from Tianshui Hong Shan Testing Machine Co., Ltd., Tianshui, China, and the specification type was YAW-30000. During the tests, displacement control was applied at a constant rate of 0.4 mm/min. Upon reduction of the load to 70% of the peak load or when the deformation of the specimen was too large to continue loading, the test was stopped.

**Figure 5:**
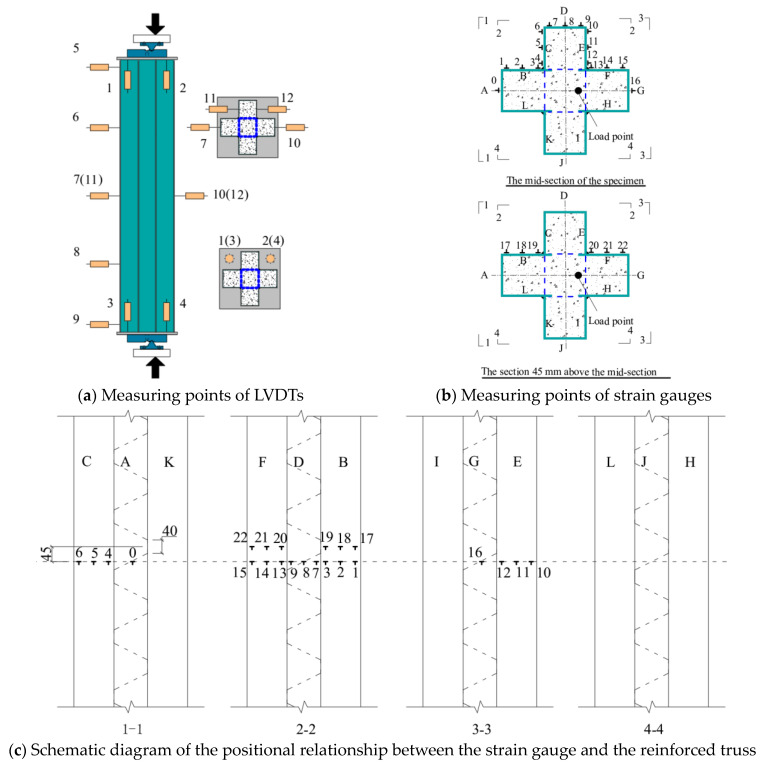
Arrangement of measuring points.

The load data of the test were collected by the testing machine. The displacement data were taken by 12 linear variable displacement transducers (LVDTs) (Figure 5a). Specifically, axial deformation between two end caps was captured using 4 LVDTs, 5 LVDTs were installed in the height direction to gauge the horizontal deflection of specimens, 2 LVDTs were used to measure the horizontal displacement at concave corners of specimens, and, in addition, a No.10 LVDT was set up to measure the change at the mid-span of the specimen. The strains of steel plate were obtained by 23 groups of strain gauges of which 17 groups were arranged on the steel tube of the middle section of the specimen, and each group contained 2 strain gauges to measure horizontal and longitudinal strains. In addition, a total of 6 groups of strain gauges were set on the steel plates No.1 and No.2 at 45 mm above the middle section of the column. The specific layout is shown in Figure 5b,c.

## 3. Test Results and Discussion

### 3.1. Failure Modes

**Figure 6:**
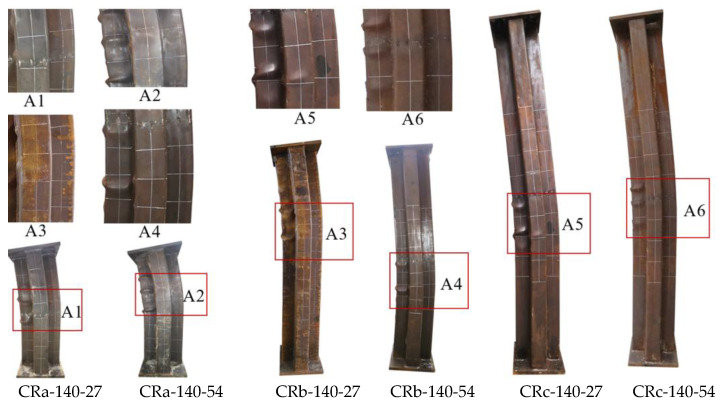
Failure phenomenon of steel tubes of truss-reinforced specimens.

Under eccentric compression, the CCFST specimens showed different failure modes with the change in slenderness ratio (Figure 6). For the short specimen CRa-140-27 with a height of 1000 mm, at the initial stage of loading, the deformation of the longitudinal and horizontal deformation were not significant. After gradually increasing the load to around 60% of the peak load, the specimen began to produce the “ZiZi” sound of the steel tube separated from concrete inside the specimen. When loaded to 95% of the peak load, the middle of the G-surface of the steel tube in the compression zone began to buckle slightly, while the overall horizontal deformation was not significant. As the load approached the peak value, slight buckling began to appear on the steel plates (F and H), the specimen showed a small bending deformation as a whole, and the horizontal deflection near the mid-section of the column gradually increased. After the peak load was reduced to 85%, the horizontal deformation was serious. There were obvious multi-wave bulging phenomena on the steel plates (G, F, E, and H) near the mid-section of the column, with the spacing of the wave peaks ranging from 120 mm to 150 mm. The short specimen CRa-140-27 showed the failure mode of the steel tube first by undergoing bulging, and then the overall bending deformation of the specimen occurred, which is a typical strength failure characteristic.

**Figure 7:**
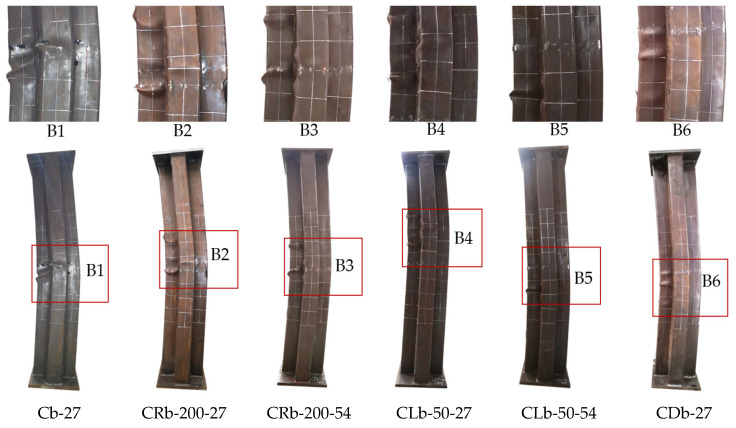
Failure phenomenon of specimens with different configuration forms.

For specimens with heights of 1800 mm and 2500 mm, the failure modes were generally close to each other (Figure 7). Taking the truss-reinforced specimen CRb-200-27 as an example, the eccentric compression test process was analyzed in detail. There was no discernible alteration in the appearance of the specimen from the initial stage of loading to 75% of a peak load, and the deflection growth was proportional to the load growth. With the continuous increase in load, the specimen began to show flexural deformation, and the change in horizontal deflection at the mid-section of column was more obvious than that of the column with a height of 1000 mm. As the peak load was approached, new plate buckling occurred 300 mm above the mid-section of the G-surface, and multiple small plate buckling occurred on the F-surface as well, with the bulge wave peaks spaced about 120 mm apart. As the displacement load increased further, the axial load gradually decreased, and the degree of bulging at the original steel plate bulge increased. When the load was reduced to 3300 kN (0.95 Nu), two new steel tube bulges were added on the E-surface, the peak of the bulge wave was staggered with the F-side, and the steel plate bulge also appeared on the D-surface at a point 70 mm above the mid-section of the column. The test was stopped when the load was reduced to 85% of the peak load, and then the truss-reinforced specimen showed a typical bending failure mode.

**Figure 8:**
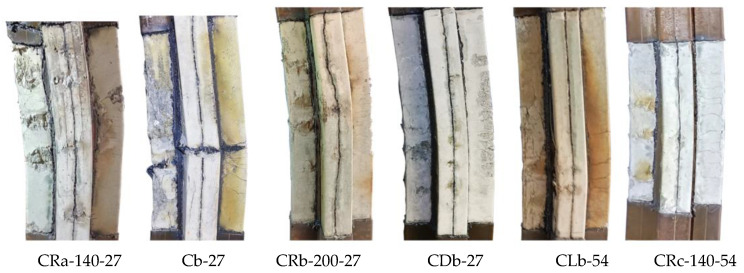
Failure phenomenon of concrete of specimens with different configuration forms.

During the test of the unstiffened specimen Cb-27, a small local buckling first appeared in the middle of the G-surface. With the increase in vertical displacement load, significant steel tube bucklings were also observed (E, F, H, and I). At the end of the test, it was observed that the steel plates at concave corners near the mid-span of the column were pulled out by the expansion force of concrete. Following the cutting of steel tube (Figure 8), it was observed that there was an obvious crushing phenomenon on the plates (F and G); at the same time, lateral cracks appeared in the concrete in the middle of the A-surface. For truss-reinforced specimen CRb-200-27, the concrete on the F-surface, the G-surface had multiple crushings along the height of the column, and the crushing area corresponds to the buckling position of the steel plate. Multiple lateral cracks were observed in the concrete of the column limb in the tensile zone at the far end of the force. Compared to the unstiffened specimen Cb-27, the lateral cracks in the concrete of specimen CRb-200-27 had a wider distribution, the number of cracks increased significantly, and the cracks were more evenly distributed. Additionally, by observing specimens with multi-cell and spiral stirrup stiffening, it was noted that the multi-cell specimen showed multiple bulging waves within 200 mm above and below the mid-span of the column, resembling the form observed in the truss-reinforced specimen. The failure mode of specimens with spiral stirrup stiffening was similar to that of unstiffened specimens, characterized by the steel tube being extruded laterally due to the expansion of concrete and a severe lateral bulging of the compressed zone’s steel tube. By comparing the phenomenon of lateral crack expansion in the tension section of the concrete on the A-surface during the failure of specimens with different configuration forms (Figure 8), it was evident that, for specimens with the same eccentricity ratio, those with reinforced truss exhibited a wider range of lateral crack expansion along the column height and smaller crack widths compared to unstiffened specimens.

**Figure 9:**
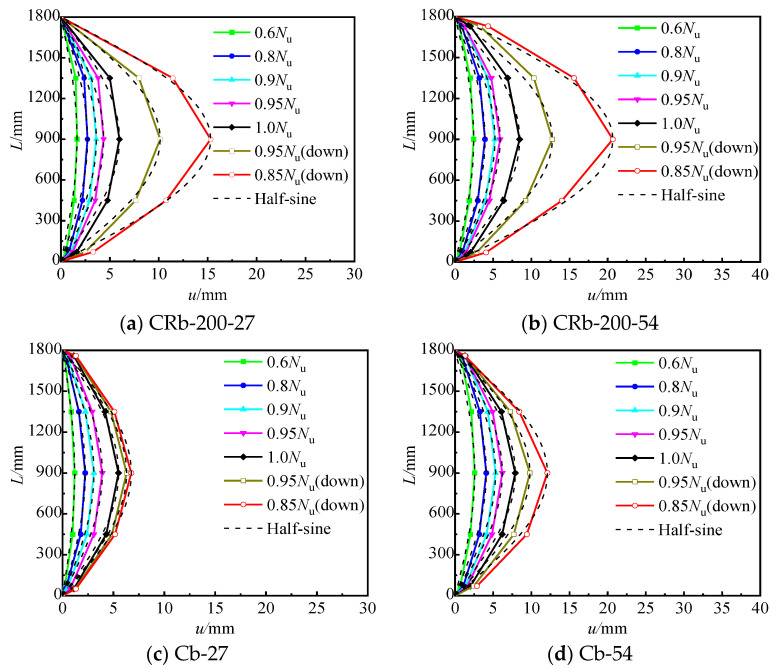
Deflection deformation curve of specimens along the height direction.

The horizontal deflection distribution curves of the specimens reflect the characteristics of horizontal deformation. In this study, five horizontal displacement gauges were installed along the height direction of the specimen. Horizontal deflection curves under different load levels were plotted using deflection data (Figure 9). The vertical axis represents the height coordinates from the bottom of the column, and the horizontal axis represents horizontal deflection at different heights from the bottom of the column. *N*_u_ is the ultimate load value of the specimen, and the “down” in brackets represents the descending segment. As shown in Figure 9, the bending deformation of all specimens generally follows a sine half-wave curve, and the development of the curves corresponds closely to the failure process of specimens. Before reaching the peak load, the horizontal deflection trends at the mid-span of all specimens were generally consistent. Upon gradual increase of the load to peak load, the differences in horizontal deflections became increasingly apparent. Compared with unstiffened specimen Cb-27, the horizontal deflection at the mid-section of specimen CRb-200-27 increased faster. When the load was reduced to 0.85 *N*_u_, the deflection at the mid-span of specimen CRb-200-27 reached 15.3 mm, which was significantly higher than the 6.75 mm of the unstiffened specimen Cb-27. When the eccentricity ratio increased to 0.15, the horizontal deflection of truss-reinforced specimen CRb-200-54 was still growing faster than that of specimen Cb-54, and the deflection reached 20.7 mm and 12.1 mm, respectively. This shows that after the stiffened measure is adopted, the deformability of the member is enhanced, and the synergistic working of the steel tube and concrete is improved after the peak load.

### 3.2. Influence of Parameters

#### 3.2.1. Configuration Forms

**Figure 10:**
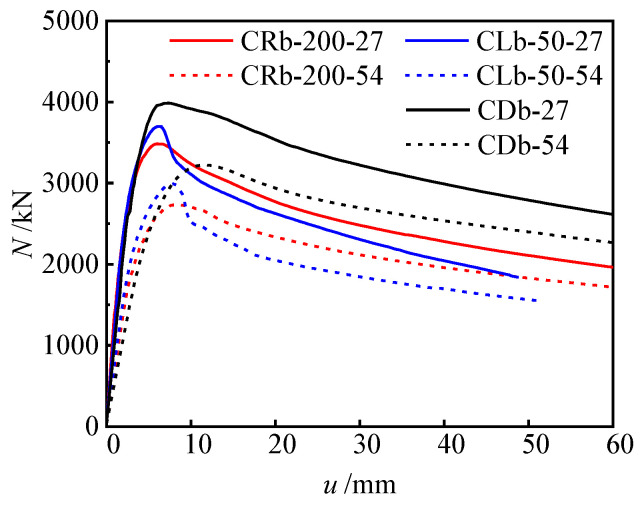
Load (*N*)–horizontal deflection (*u*) at 1/2 height relationship curves of different configuration forms.

In this section, the load (*N*)–horizontal deflection (*u*) at 1/2 height relationship curves of different configuration forms (spiral stirrup stiffening, multi-cell, and reinforced truss stiffening) are plotted (Figure 10) to compare their flexural capacity and ductility performance. For the specimens with an eccentricity ratio of 0.08, in the elastic phase, the load (*N*)–horizontal deflection (*u*) at 1/2 height relationship curves exhibit linear development with increasing load, and the stiffness development of the three types of specimens is essentially consistent. Upon entering the elastic–plastic phase, the stiffness of the load (*N*)–horizontal deflection (*u*) at 1/2 height relationship curves for specimens CRb-200-27 (reinforced truss), CLb-50-27 (spiral stirrup stiffening), and CDb-27 (multi-cell) slowly decreases. At peak load, specimen CRb-200-27 has a lower bearing capacity, while specimens CLb-50-27 and CDb-27 exhibit increases of 6.2% and 14.4%, respectively, compared to specimen CRb-200-27. After reaching peak load, the load–deflection curve of specimen CLb-50-27 (spiral stirrup stiffening) rapidly descends, and it exhibits a ductility coefficient of 2.79, indicating poorer ductility. In contrast, specimens CRb-200-27 and CDb-27 achieve ductility coefficients of 4.88 and 5.49, respectively, showing improvements of 74.9% and 96.8% compared to specimen CLb-50-27, demonstrating better ductility. Spiral stirrup provides a certain amount of confinement to the internal concrete, which can increase the bearing capacity of the hooped concrete. But it has no obvious restriction effect on the steel at the concave corner, and then the steel tube and the concrete at the corner are separated, which is the primary reason for the worse ductility of specimens with spiral stirrup stiffening compared to those with reinforced truss and multi-cell forms.

When the eccentricity ratio increased to 0.15, the load (*N*)–horizontal deflection (*u*) at 1/2 height relationship curves of the steel-bar truss stiffening form, the spiral stirrup stiffening, and the multi-cell specimens were basically consistent with the above laws. The ductility coefficients of CRb-200-54 and CDb-54 were 4.46 and 4.05, respectively, while the ductility coefficient of CLb-50-54 was only 2.18, which indicated that the truss-reinforced specimen showed better ductility, even close to that of the specimen of the multi-cell form. In addition, the steel ratio of the reinforced truss form was about 9.8%, while that of the multicavity form was 11.6, and the former reduced the steel ratio by 1.8% in comparison with the latter, which saves steel.

#### 3.2.2. Reinforced Truss Node Spacings

**Figure 11:**
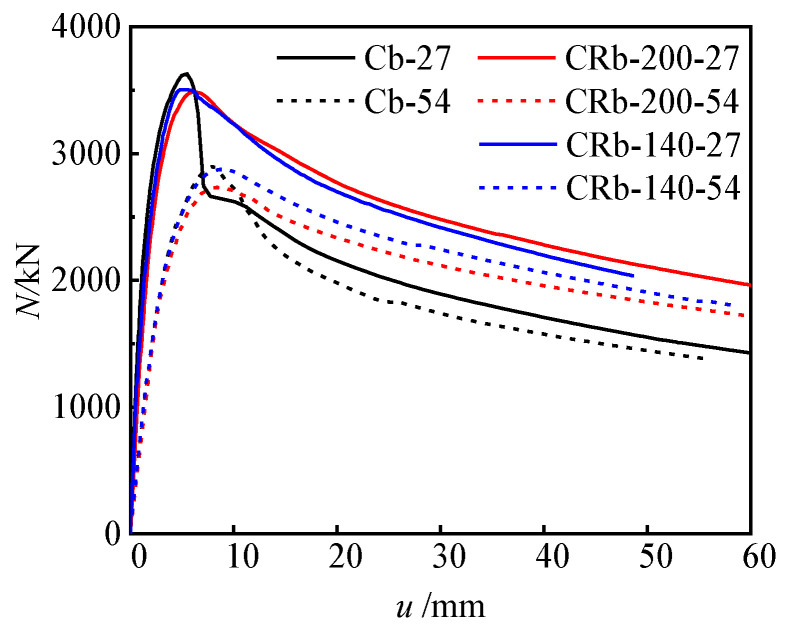
Load–deflection curves of typical specimens.

When the reinforced truss node spacing of the specimen tends to infinity, its mechanical properties approximate those of the unstiffened specimen. To investigate the influence of the reinforced truss node spacing on the mechanical properties of components, this section compares and analyzes the load (*N*)–horizontal deflection (u) at 1/2 height relationship curves of specimens with node spacings of infinity (unstiffened), 200 mm, and 140 mm (Figure 11). During the initial loading phase up to approximately 0.7 *N*_u_, specimens with the same eccentricity exhibited similar stiffness development trends, with deflection growth proportional to the increase in load. As the load further increased towards the peak load, the specimens entered the elastoplastic stage, where the load (*N*)–horizontal deflection (*u*) at 1/2 height relationship curves exhibited nonlinear development, but the differences between the curves were not significant. When loaded to the peak load, the peak loads of the unstiffened specimen Cb-27, the specimen CRb-200-27 with a node spacing of 200 mm, and the specimen CRb-140-27 with a node spacing of 140 mm were 3628 kN, 3483 kN, and 3504 kN (Table 1), respectively, with a difference of no more than 5% between them. When the eccentricity increased to 54 mm, the peak load differences of these three types of specimens remained within 6%. Therefore, it can be inferred that changes in the reinforced truss node spacing had a minor impact on the eccentric compression bearing capacity of the specimens. However, once the load (*N*)–horizontal deflection (*u*) at 1/2 height relationship curves of the specimens entered the descending segment, the differences between different specimens became significant. Specifically, the curve of the unstiffened specimen experienced a steep drop, while the curves of the stiffened specimens with node spacings of 200 mm and 140 mm exhibited a gradual descending trend, demonstrating good deformability. Finally, the ductility coefficients of specimens Cb-27, CRb-200-27, and CRb-140-27 were calculated as 2.67, 4.88, and 4.84, respectively (Table 1). When the eccentricity increased to 54 mm, the ductility coefficients of these three types of specimens decreased to 2.55, 4.46, and 4.35, respectively. Therefore, the introduction of steel truss stiffening significantly affected the ductility of the specimens, with an improvement of over 70%. When the reinforced truss node spacing was reduced from 200 mm to 140 mm, the ductility improvement of the specimens was not significant, further indicating that a node spacing of 200 mm is sufficient to achieve significant enhancement in the ductility of the component.

#### 3.2.3. Slenderness Ratios and Eccentricity Ratios

**Figure 12:**
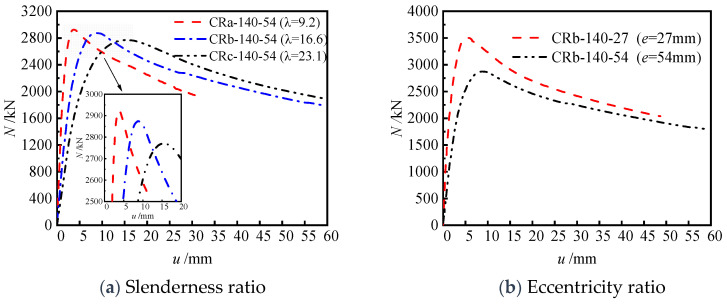
Load (*N*)–horizontal deflection (*u*) at 1/2 height relationship curves of specimens with different slenderness ratios and eccentricity ratios.

To investigate the influence of the slenderness ratio on the eccentric compression behavior of truss-reinforced specimens, a comparative analysis was conducted on the load (*N*)–horizontal deflection (*u*) at 1/2 height relationship curves of specimens with different slenderness ratios (*λ* = 9.2, 16.6, 23.1) (Figure 12a). In the initial loading stage, specimens with larger slenderness ratios exhibited lower initial stiffness. While the load increased to the peak load, specimens with larger slenderness ratios showed greater horizontal deflection at the mid-span and smaller peak load. Once the specimen curves entered the descending segment, the curves of specimens with different slenderness ratios all declined relatively smoothly, indicating the good ductility of the truss-reinforced specimen. Specifically, the ductility coefficients of specimens CRa-140-54 (*λ* = 9.2), CRb-140-54 (*λ* = 16.6), and CRc-140-54 (*λ* = 23.1) reached 6.02, 4.35, and 4.22, respectively. Additionally, a comparative analysis was also conducted on the load (*N*)–horizontal deflection (*u*) at 1/2 height relationship curves of specimens with different eccentricities (*e* = 27 mm and 54 mm) (Figure 12b). It was found that with the increase in eccentricity, the elastic segment became shorter, the bearing capacity decreased, and the curve declined more smoothly after reaching the peak load.

### 3.3. Enhancing Mechanism of Steel-Bar Truss

#### 3.3.1. Stress of Steel Tube at Concave Corners

**Figure 13:**
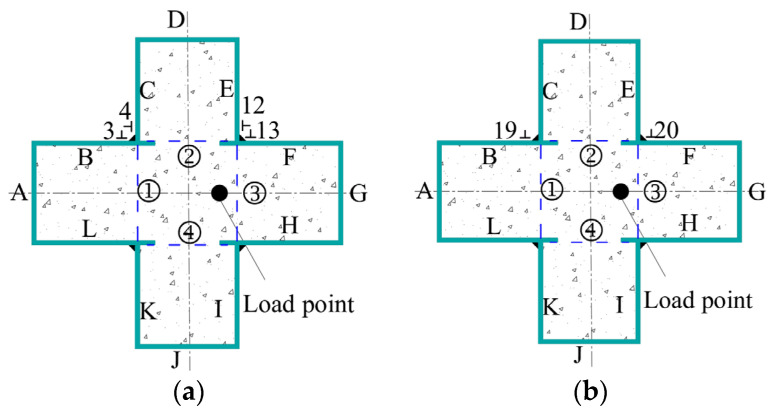
Schematic diagram of the measuring points at the concave corner of the truss-reinforced specimen. (**a**) The measuring points at the mid-section of the specimen. (**b**) The measuring points 45 mm away from the mid-section along the height of the specimen.

To further elucidate the restraining effect of the reinforced truss on the steel tube at the concave corner and the confinement mechanism of the steel tube on concrete for the CCFST column, the strains of the steel plates at concave corners of unstiffened and stiffened specimens were monitored. The measuring points of the unstiffened specimens were arranged similarly to the strain gauges at the mid-span of the truss-reinforced specimen (Figure 13a). Additionally, based on the setting of the reinforced truss along the height, strain monitoring was conducted at another critical section (Figure 13b). Ultimately, the strain data were converted into stress using an elastoplastic analysis method [38], yielding the stress curves of the steel tubes under eccentric compression. *σ*_v_ and *σ*_h_ are the vertical and horizontal stresses of the steel plate, respectively. *σ*_z_ is not the stress of the steel plate, but rather the equivalent stress obtained from the calculation of vertical and horizontal stresses, $\sigma_{z}=\sqrt{\sigma_{h}^{2}+\sigma_{v}^{2}-\sigma_{h}\sigma_{v}}$.

**Figure 14:**
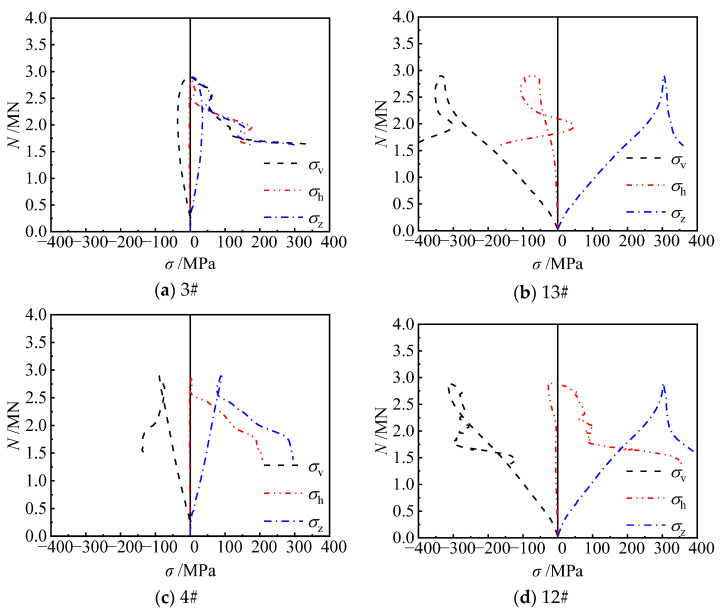
Stress of steel plate at the concave corner of specimen Cb-54.

For unstiffened specimen Cb-54 (Figure 14), before the peak load, steel plates at the concave corner were mainly under longitudinal compressive stresses, the horizontal stress was small, and the synergistic action between the steel tube and the concrete at the concave corner was not obvious. After the peak load, the horizontal stress development at each measuring point was still slow, and when the specimen was destroyed, the horizontal tensile stress at points 3#, 4#, and 12# was 1.9 MPa, 22.6 MPa, and 66.2 MPa, respectively, which was in line with the general rule that the horizontal stress of steel plate had a small increase after the extrusion of the side wall of the steel tube by concrete expansion under compressive loads. As for measuring point 13#, it was subjected to horizontal and longitudinal two-way compressive stress, and the confinement effect on concrete was weaker. This shows that the steel plate of the unstiffened specimen can only resist the horizontal deformation of concrete through its own stiffness, and lacks effective horizontal confinement to concrete, which cannot effectively exert the combined effect of the steel tube and concrete.

**Figure 15:**
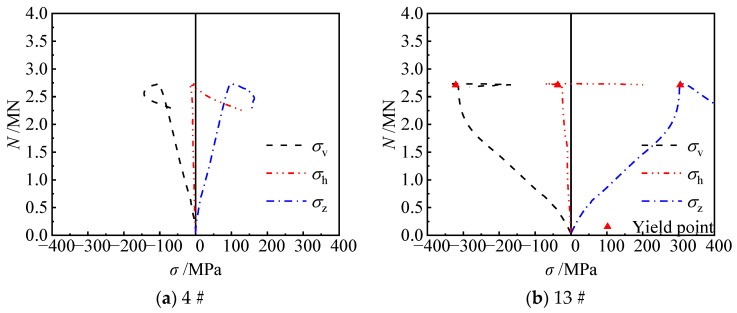
Stress of steel plate at the concave corner of specimen CRb-200-54.

For specimen CRb-200-54 (*s* = 200 mm, *e* = 54 mm) (Figure 15), before the peak load, the development of horizontal stresses at concave corners of the steel tube was not obvious, and the effect of the steel bars was not demonstrated. At peak loads, the horizontal stresses gradually began to develop. When the load decreased to 2713 kN (0.99 *N*_u_), the horizontal tensile stress at point 13# increased rapidly to 200.0 MPa; meanwhile, the horizontal tensile stress at point 4# also increased to 98.1 MPa when the load decreased to 2322 kN (0.85 *N*_u_). This shows that the addition of diagonal bars can effectively restrict the buckling of the steel tube at concave corners, enhance the confinement of concrete by the steel tube, and realize the synergistic working of the steel tube and concrete.

**Figure 16:**
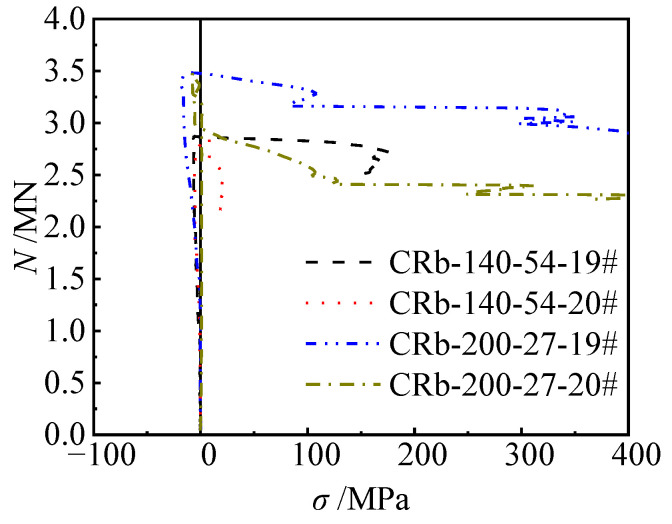
Horizontal stress profile at the concave corner 45 mm above the mid-section.

In order to further illustrate the development of the stresses in the steel tube at concave corners along the column height direction, a section 45 mm away from the mid-section of the specimen was selected as the critical section. By observing the horizontal stress curves of steel plates of specimen CRb-200-27 and specimen CRb-140-54 (Figure 16), it was found that before reaching the peak load, the horizontal stress at points 19# and 20# developed slowly. During the process from peak load to specimen failure, the horizontal stress at point 19# developed rapidly, with the maximum horizontal tensile stress reaching 356 MPa and 176 MPa for specimens CRb-200-27 and CRb-140-54, respectively. The tensile stress at point 20# gradually appeared only after the specimen was damaged. The primary reason is that at point 19#, the steel plate was directly subjected to the in-plane tensile force generated by the diagonal bar ②. As for point 20#, the point was located in the position between the two adjacent welding points of the diagonal bar ②, and the diagonal bar did not act directly on the steel plate at this point, so that the influence of the steel bars on the internal tension in the steel plate was small, and was only visible when the specimen underwent significant damage and deformation.

**Figure 17:**
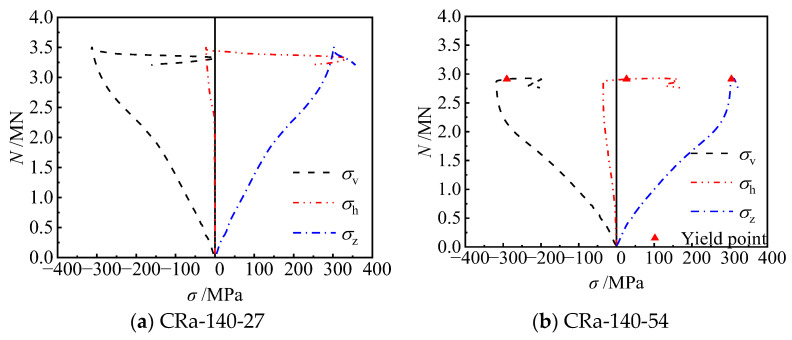
Stress of point 13# at the concave corner of specimens.

When the slenderness ratio of the specimens was reduced to 9.2 (Figure 17), for the truss-reinforced specimen CRa-140-27 (*λ* = 9.2, *e* = 27 mm), prior to peak load, the horizontal tensile stress at point 13# developed slowly; the horizontal tensile stress began to develop rapidly and had grown to 332 MPa when the load decreased to 0.94 *N*_u_. When the eccentricity ratio increased to 0.15, the horizontal tensile stresses at point 13# of specimen CRa-140-54 (*λ* = 9.2, *e* = 54 mm) increased gradually. When the specimen was destroyed, the horizontal tensile stress at point 13# reached 168 MPa.

In summary, the reinforced truss has a certain restraining effect on the steel tubes at concave corners of the member. Steel-bar trusses arranged along the bending direction of the member can effectively enhance the confinement of concrete by the steel tube, thereby improving the overall eccentric compression behavior of the member.

#### 3.3.2. Longitudinal Strain Distribution of Steel Tube

**Figure 18:**
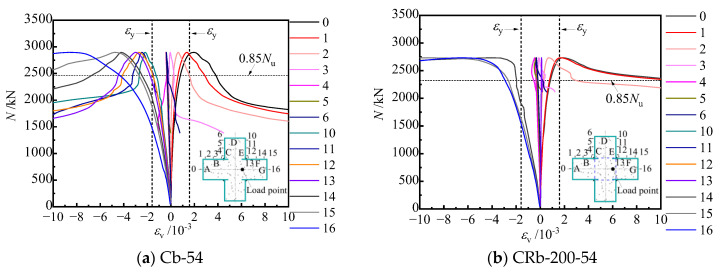
Longitudinal strain curve of steel tube with mid-section.

**Figure 19:**
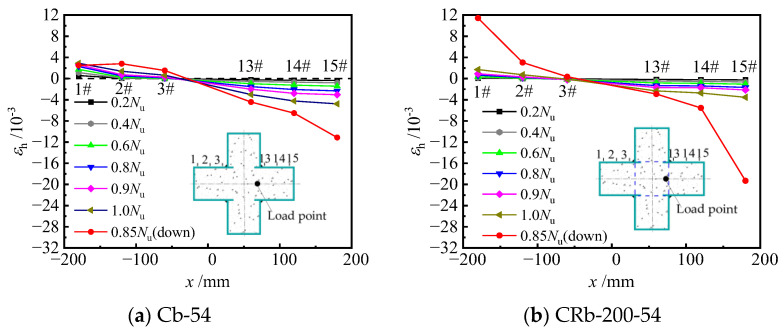
Longitudinal strain distribution at the mid-span.

The damage of the specimens mainly occurred near the column 1/2 height and the bending moment of the specimens and the longitudinal strain values of the steel tubes were the largest here; therefore, the longitudinal strains at this height are discussed. The longitudinal strain load distributions of steel tubes for typical specimens Cb-54 and CRb-200-54 are shown in Figure 18 and Figure 19, where positive strains are tensile strains and negative strains are compressive strains.

Figure 18 gives the longitudinal strain relationship curves for the steel tube at the mid-section of the specimen, with a yield strain *ε*_y_ of 1590 microstrains. The curve development trend corresponds to the specimen’s failure process. In the early stages of loading, the steel tube strain increasedlinearly with increasing load. As loading continued, both the positive and negative bending of the specimen entered the plastic stage starting from the specimen’s edge. Firstly, the longitudinal strain of the G-surface at the edge of the compression zone began to increase rapidly, and reached the yield strain first, and then the strain at points 15~10 also reached the yield strain in turn (Figure 18). When the load reached a peak, the tensile steel plate A-surface at the distal end of the two typical specimens Cb-54 and CRb-200-54 also reached the yield strain almost at the same time; the longitudinal stresses in the column mid-section of specimens Cb-54 and CRb-200-54 varied differently upon peak load. The longitudinal strain curves of truss-reinforced specimen CRb-200-54 dropped more gently as compared with that of Cb-54, with a larger increase in longitudinal strains in the B-surface of the tensile plate and the F-surface of the compressive steel plate when the load of the specimen decreased to 0.85 *N*_u_. The longitudinal strain curves of the compression plate F-surface decreased more gently, and the longitudinal strain of the tensile A-surface had increased greatly when the load of the specimen decreased to 0.85 *N*_u_. It can also be observed more obviously through Figure 19 that the longitudinal strain distribution patterns of typical specimens were basically the same in different load stages before a peak load and were in line with the plane section assumption. Only after a peak load did the longitudinal strains in the tensile and compressive zones of truss-reinforced specimen CRb-200-54 have a large increase, and when the specimen was loaded up to 0.85 *N*_u_ (down), the steel plate’s No.0 tensile zone and No.16 compressive zone reached 11,427 and 19,308 microstrains, respectively, which indicates that the configuration of reinforced truss can better improve the flexural deformability of the member.

#### 3.3.3. Horizontal Strain Distribution

**Figure 20:**
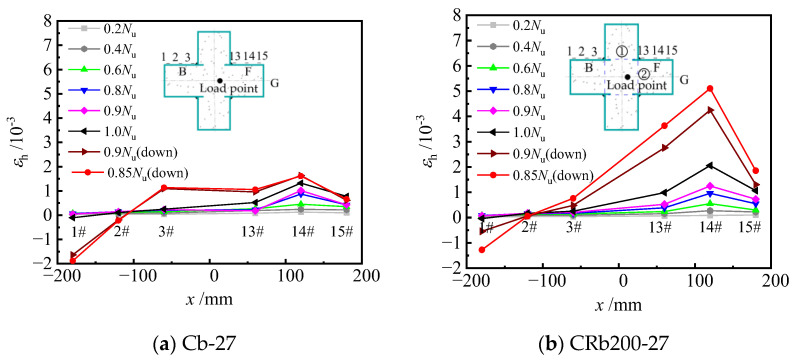
Horizontal strain distribution at the mid-span.

Figure 20 illustrates the distribution of horizontal strains along the cross-sectional height of typical specimens under different load levels. It can be observed that before loading to 0.6 *N*_u_, the horizontal strains along the cross-section height of the specimen exhibited an approximately linear distribution. As the load increased to the peak load, the horizontal tensile strains at measuring points 13#, 14#, and 15# increased gradually, the horizontal strains at measuring points 1#, 2#, and 3# changed less, while the horizontal tensile strain at measuring points 13#, 14#, and 15# increased to some extent. Compared with unstiffened specimen Cb-27, the horizontal strain of truss-reinforced specimen CRb200-27 increased more during loading to 0.8 *N*_u_ to 1.0 *N*_u_. When loaded to the peak load, the horizontal tensile strains continued to increase in the compression zone at the measuring points 13#, 14#, and 15#, and the increase in specimen CRb200-27 was particularly significant. The main reason for the above law is that under eccentric compression, the longitudinal strain on the B-surface is smaller compared to that on the F-surface, and the horizontal deformation of the concrete in the column limb of the compression zone on the side of the F-surface is larger and squeezes the steel plate in two directions perpendicular to F-surface and G-surface, which leads to an increase in the horizontal strain at points 13#, 14#, and 15#. Meanwhile, the out-of-face bulging deformation occurred at the F-surface, which caused the horizontal strain at point 14# to be larger compared with points 13# and 15#. Specimen CRb200-27 had a diagonal bar ① welded near measuring point 13#. The diagonal bar ① acted on the F-surface of the steel plate to prevent the F-surface from being pulled out in the direction parallel to the F-surface. As a result, compared to the unstiffened specimen Cb-27,the addition of the diagonal bar ① increased the horizontal strain of the steel plate F-surface. Especially post peak load, the deformation of concrete was further intensified and then squeezed the steel plate G-surface and F-surface, whereas the steel bar ② limited the out-of-face displacement of the steel plate F-surface at the concave corner. Accordingly, the confinement effect of steel tube on concrete of the column limb was enhanced, especially after the peak load.

## 4. Comparison with Existing Design Codes

Currently, there are various design codes to calculate the bearing capacity of composite members under combined compression and bending in China and abroad, and there is a large difference between calculation methods. Domestically, there are GB50936 [39], CECS159 [40], etc., and foreign design codes on CFST include Eurocode-4 [41], AISC360 [42], and so on. This paper focuses on the relevant calculations in the four design codes mentioned above.

The solution to GB50936 consists of two terms relating to axial forces and moments.

(1) $$-\frac{N}{2.17N_{u}}+\frac{\beta_{m}M}{M_{u}(1-0.4N/N_{E}^{\prime })}\leq 1\;\;\frac{N}{N_{u}}<0.255$$

(2) $$\frac{N}{N_{u}}+\frac{\beta_{m}M}{1.5M_{u}(1-0.4N/N_{E}^{\prime })}\leq 1\;\;\frac{N}{N_{u}}\geq 0.255$$

where *N*_u_ is the stable load of composite columns under axial force; *M*_u_ is the load-carrying capacity in bending; *β*_m_ is the equivalent bending moment coefficient; and *N*′_E_ is the Euler load of the column considering the partial coefficient of the material.

The solution to CECS159 is provided by

(3) $$\frac{N}{\varphi_{x}N_{u}}+(1-\alpha_{c})\frac{\beta_{m}M_{x}}{(1-0.8\frac{N}{N_{\mathrm{Ex}}^{\prime }})M_{u}}\leq 1$$

(4) $$\frac{\beta_{m}M_{x}}{(1-0.8\frac{N}{{N^{\prime }}_{\mathrm{EX}}})M_{u}}\leq 1$$

(5) $$\frac{N}{\varphi_{y}N_{0}}+\frac{\beta_{m}M_{x}}{1.4M_{u}}\leq \frac{1}{\gamma_{0}}$$

where *γ*_0_ is the configuration importance coefficient, 1.0 in the case of this study; *φ*_x_ and *φ*_y_ are the stability coefficients under the axial compression in the plane of bending moment action and out of the plane of bending moment action, respectively; *N*_0_ is the load-carrying capacity in compression; *α*_c_ is the percentage of load-carrying capacity shared by concrete; and *N*′_EX_ is the Euler load of the column considering the partial coefficient of the material.

**Figure 21:**
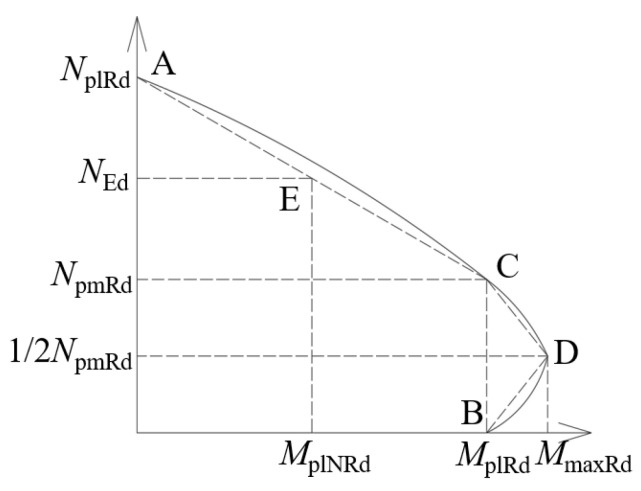
*N*-*M* interaction curve.

The *N*-*M* curve (Figure 21) in Eurocode-4 can be simplified into three straight lines for the design value of the plastic compression capacity of the section and for the design value of the plastic bending capacity of the section. *M*_maxRd_ is the maximum design plastic resistance moment, *N*_pmRd_ is the design axial force of the concrete section, and *M*_Ed_ and *N*_Ed_ are the axial bending moment and the axial force, respectively.

Composite members should satisfy the following conditions under compression–bending combination:

(6) $$\frac{M_{\mathrm{Ed}}}{M_{\mathrm{plNRd}}}=\frac{M_{\mathrm{Ed}}}{\mu_{d}M_{\mathrm{plRd}}}\leq \alpha_{m}$$

(7) $$\frac{N_{\mathrm{Ed}}}{\chi N_{\mathrm{plRd}}}\leq 1$$

where *M*_plRd_ and *M*_plNRd_ are the plastic resistance moments of the cross-section without considering the axial force and considering the axial force, respectively; *M*_Ed_ is the greatest moment in the member; *α*_m_ is a coefficient related to the strength grade of the steel, which is taken to be 0.9 for steel grades between Q235 and Q345; and *χ* is a reduction factor related to the slenderness ratio.

According to AISC360, composite sections are divided into noncompact, compact, and slender based on the width-to-thickness ratio of the steel section. The analysis methods for these different types of composite sections vary slightly. In this study, all the cross-sections considered are classified as compact sections. The solution for compact sections is then given by:

(8) $$\frac{N}{N_{u}}+\frac{8}{9}\frac{M}{M_{u}}\leq 1\;\;\frac{N}{N_{u}}\geq 0.2$$

(9) $$\frac{N}{2N_{u}}+\frac{M}{M_{u}}\leq 1\;\;\frac{N}{N_{u}}<0.2$$

Code AISC360 is similar to CECS159, which does not consider the confinement effect of concrete by the steel tube.

In this paper, the four calculation methods mentioned in the previous section were used to obtain the eccentric compression bearing capacity of the members, and the calculations were compared with the test results (Table 3), so as to compare the accuracy of the calculation methods in each code. It should be noted that the relevant parameters in the code use the design value, while this article uses the standard value. In addition, due to the initial defects of specimen CRb-200-0 or an error during the test, bending deformation occurred during the loading process, which led to the low bearing capacity of the test. Therefore, when comparing with the existing design specifications, the data of the specimen were not considered.

With the increase in the eccentricity, there is a tendency to increase the ratio of calculated to experimental values of EC4, and in contrast, the ratio of the calculated value of GB50936 to the test value has a tendency to decrease. By comparing the calculation results of CECS159, it can be found that the calculated values are smaller than the test values, which are on the safe side, with an mean deviation within 7% and a small dispersion.

In summary, the results obtained using EC4, GB50936, and CECS 159 were in perfect correspondence with the test values. The mean values of the three methods were 1.024, 1.014, and 0.936 and the coefficients of variation were 0.049, 0.039, and 0.018, respectively, which indicate high stability and accuracy in predicting the bearing capacity. Therefore, within the parameter range of this paper, it is recommended to adopt the calculation methods in EC4, GB50936, and CECS 159 to calculate the eccentric compression bearing capacity of truss-reinforced CCFST columns.

## 5. Conclusions

This paper presents a detailed investigation into the eccentric compression tests on the CCFST columns. The experimental procedures and observations are thoroughly described, focusing on the eccentric compression behavior of truss-reinforced columns. The obtained results include the load (*N*) versus horizontal deflection (*u*) curves, load–stress curves, and load–strain curves for the steel tube. Comparative analyses are conducted for various test parameters including the reinforced truss node spacing, slenderness ratio, and eccentricity ratio. Based on the study, the following conclusions are drawn:

(1) The reinforced truss is effective in enhancing concrete confinement by the steel tube, leading to significantly improved eccentric compression behavior for the CCFST. The failure mode of the steel tube experienced multiple-wave buckling rather than single-wave buckling for the truss-reinforced CCFST columns, and the concrete experienced multi-section crushing rather than single-section crushing.
(2) The reinforced truss could significantly improve the deformation ability of the eccentrically compressed CCFST column, and the ductility coefficient was increased by 70~80% compared with the unstiffened column, which could be close to that of the multi-cell member. In addition, the ductility was not changed much when the reinforced truss node spacing was reduced from 200 mm to 140 mm, and it is concluded that the ductility requirement for the member could be satisfied when the node spacing is 200 mm.
(3) The addition of the reinforced truss can restrict the out-of-plane displacement at concave corners of the steel tube, enhance the confinement of the steel tube to the core concrete, improve the overall eccentric compression behavior of the member, and enhance the synergistic working of the two materials, steel tube and concrete.
(4) By comparing the design code methods and test results, it is concluded that results obtained using EC4, GB50936, and CECS 159 are in perfect correspondence with the test values. Therefore, within the range of parameters in this paper, it is recommended to adopt the calculation methods in Chinese codes GB50936 and CECS159, and in Eurocode 4 to calculate the eccentric compression bearing capacity of truss-reinforced CCFST columns.

## Figures and Tables

**Table 1:** Main parameters of specimens.

No	Specimen	Axial Length	Slenderness Ratio	Stiffener Type *s* or *d* (mm)	Steel Ratio *α* (%)	Load Eccentricity	Bearing Capacity	Ductility Coefficient [34]
		*L* (mm)	*λ*			*e* (mm)	*N*_u_ (kN)	*μ*
1	CRa-140-0	1000	9.2	140	9.85	0	4600	——
2	CRa-140-27	1000	9.2	140	9.85	27	3544	6.85
3	CRa-140-54	1000	9.2	140	9.85	54	2926	6.02
4	CRb-140-0	1800	16.6	140	9.85	0	4602	——
5	CRb-140-27	1800	16.6	140	9.85	27	3504	4.84
6	CRb-140-54	1800	16.6	140	9.85	54	2874	4.35
7	CRb-200-0	1800	16.6	200	9.77	0	3753	——
8	CRb-200-27	1800	16.6	200	9.77	27	3483	4.88
9	CRb-200-54	1800	16.6	200	9.77	54	2733	4.46
10	CRc-140-0	2500	23.1	140	9.85	0	4483	——
11	CRc-140-27	2500	23.1	140	9.85	27	3473	4.10
12	CRc-140-54	2500	23.1	140	9.85	54	2770	4.22
13	Cb-27	1800	16.6	No stiffening	9.27	27	3628	2.67
14	Cb-54	1800	16.6	No stiffening	9.27	54	2897	2.55
15	CLb-50-27	1800	16.6	50	9.66	27	3698	2.79
16	CLb-50-54	1800	16.6	50	9.66	54	2990	2.18
17	CDb-27	1800	17.5	Multi-cell form	11.61	27	3986	5.49
18	CDb-54	1800	17.5	Multi-cell form	11.61	54	3223	4.05

Note. *λ* = *L*/*i*, where *L* and *i* are the height of the specimen and the radius of gyration of the specimen, respectively [35]. *i* = (*E*_s_*I*_s_ + 0.2 *E*_c_*I*_c_)^1/2^/(*E*_s_ *A*_s_ + 0.2 *E*_c_*A*_c_)^1/2^, where *I*_s_ and *I*_c_ are the moment of inertia of steel and concrete, respectively, and *E*_s_ and *E*_c_ are the modulus of elasticity of steel and concrete, respectively.

**Table 2:** Mechanical properties of steel.

Type	*f*_y_ (MPa)	*f*_u_ (MPa)	*E*_s_ (GPa)	*μ*_s_
Steel plate (*t* = 3.90 mm)	304.2	444.3	208	0.281
Steel bar (*φ* = 7.24 mm)	539.4	606.0	206	0.270

**Table 3:** Comparison of test results with calculations from relevant codes.

Specimen	*N*_tesk_ /kN	*N*_EC_ /kN	*N*_AISC_ /kN	*N*_GB_ /kN	*N*_CECS_ /kN	$\frac{N_{\mathrm{EC}}}{N_{\mathrm{test}}}$	$\frac{N_{\mathrm{AISC}}}{N_{\mathrm{test}}}$	$\frac{N_{\mathrm{GB}}}{N_{\mathrm{test}}}$	$\frac{N_{\mathrm{CECS}}}{N_{\mathrm{test}}}$
CRa-140-0	4600	4415	3989	4893	4284	0.960	0.867	1.064	0.931
CRa-140-27	3544	3608	2500	3546	3322	1.018	0.705	1.001	0.937
CRa-140-54	2926	3050	1820	2780	2714	1.042	0.622	0.950	0.928
CRb-140-0	4602	4399	3941	4870	4220	0.956	0.856	1.058	0.917
CRb-140-27	3504	3595	2481	3529	3273	1.026	0.708	1.007	0.934
CRb-140-54	2874	3040	1809	2768	2677	1.058	0.629	0.963	0.931
CRb-200-27	3483	3595	2481	3529	3273	1.032	0.712	1.013	0.940
CRb-200-54	2733	3040	1809	2768	2677	1.112	0.662	1.013	0.980
CRc-140-0	4483	4315	3876	4836	4134	0.963	0.865	1.079	0.922
CRc-140-27	3473	3527	2455	3517	3208	1.016	0.707	1.013	0.924
CRc-140-54	2770	2982	1796	2752	2628	1.077	0.648	0.994	0.949
Mean value						1.024	0.726	1.014	0.936
Coefficient of variation						0.049	0.129	0.039	0.018

## Data Availability

All data included in this study are available from the corresponding author upon request.
